# Hospitals for Consumption

**Published:** 1891-01-03

**Authors:** 


### HOSPITALS FOR CONSUMPTION.

**Brompton Hospital for Consumption.**—Since its foundation in 1841, no fewer than about 433,520 patients have been relieved altogether. This hospital is well known as one where the patients are well cared for, and every effort is made to brighten their clouded lives. There is a Samaritan Fund connected with hospital, for the aid of poorer patients, and the Sea-side Fund gives help when needful, that a patient may be sent to one of the convalescent homes on the south coast, and maintained there should they benefit by the change. Fresh subscriptions are specially needed to maintain the new building which has recently been added to the hospital, making the total accommodation 321 beds. Secretary, Mr. H. Dobbin; Matron, Miss F. Abbott.

**North London Hospital for Consumption.**—This institution was thoroughly reorganised and remodelled some years ago, and, thanks to the devotion of Mr. B. A. Lyon, the chairman, it has become one of the most useful of its class. Secretary, Mr. Lionel Hill, M.A.; Matron, Miss Blaxland.

**City of London Hospital for Diseases of the Chest,** Victoria Park, E.—The average expenditure is about £10,000, towards which only £370 of the income assured. If the public could be made to realise that the late Dr. Peacock did much of his invaluable work as physician to this hospital, funds would certainly flow into its exchequer. Secretary, Mr. T. Storrar-Smith; Matron, Miss H. G. Hetherington.

**Royal Hospital for Diseases of the Chest,** City Road, E.C.—Three hundred and fifty-eight in-patients and 8,831 out- patients were relieved in 1889. Secretary, Mr. John Harrold; Matron, Miss M. L. Smith.

**Royal National for Consumption,** Ventnor.—This institution provides for a well-re ognised want by maintaining some 150 beds on the *separate* principle at Ventnor, which are mainly occupied by metropolitan cases. Secretary, Mr. E. Morgan, 34, Craven Street, W.C.; Lady Superintendent, Miss Moore.

